# The effect of different pulp capping methods on the intrapulpal temperature when using light-cured procedures

**DOI:** 10.4317/jced.59779

**Published:** 2022-08-01

**Authors:** Dayane Oliveira, Mateus-Garcia Rocha, Panagiotis Zoidis, Patricia Pereira, Ana-Paula Ribeiro

**Affiliations:** 1 Center for Dental Biomaterials, Department of Restorative Dental Sciences, College of Dentistry, University of Florida, 1395 Center Drive, Gainesville, FL, 32610

## Abstract

**Background:**

To evaluate the intra-pulpal temperature during different direct pulp capping methods and light-cured procedures.

**Material and Methods:**

Class I preparations 5 mm long, 4 mm wide and 4 mm deep were performed in extracted third molars, leaving 0.5 mm of dentin at the pulpal floor with a 1 mm diameter of pulp exposure. Teeth were placed in a customized oral cavity chamber simulator in which the initial temperature was standardized at 36oC. The overall temperature variations (oC) in the pulp chamber during the light-activation processes were recorded live using an infrared camera (FLIR ONE PRO, FLIR Systems). The liners and bases evaluated were: Dycal (Dentisply), TheraCal LC (Bisco), Biodentin (Septodent), Vitrebond Plus (3M/ESPE), and Fuji IX GP (GC), followed by restoration with a bulk fill composite (EvoCeram Bulk Fill, Ivoclar Vivadent). All light-activation procedures were performed with the VALO Grand (Ultradent) light-curing unit. A power analysis was conducted to determine the sample size to provide a power of at least 0.8 with α=0.05. Statistical analyses were performed using ANOVA and Tukey’s test for multiple comparisons.

**Results:**

The intrapulpal temperature increased above a 10oC to 20oC threshold difference for all liners and bases that were light cured. When added as second layers, neither of those could provide thermal insulation following additional light-activated procedures (*p*=0.25). The higher the number of procedures requiring light-activation, the longer the pulp temperature remained in those increased temperature thresholds.

**Conclusions:**

For direct pulp capping procedures, a reduced number light activation procedures should be indicated to reduce the time intra-pulpal temperature rises above a 10oC threshold.

** Key words:**Liner, base, calcium hydroxide, glass ionomer, dental adhesive, bulkfill composite.

## Introduction

Although regenerative techniques are being developed to reestablish pulp vitality, the use of biocompatible dental materials is still the most used technique for the direct pulp capping procedure. The ideal properties for these dental materials include the ability to stimulate undifferentiated cells in order to form a reparative dentin bridge. It may also include other properties that will allow the protection of the pulp against chemical, bacterial and thermal challenges (1). With the development of new dental pulp capping materials, the difference betweenbases and liners can be conflicting. While bases are primary used to replace missing dentin and serve as protective barriers (2-5), liners aim to protect the pulp and promote the formation of reparative dentin bridge (2,6). However, some manufacturers and research studies have characterized this last group of materials as thermal insulators (7,8).

To define a dental material as a thermal insulator, two properties need to be considered: thermal conductivity and thermal diffusivity. Thermal conductivity is a measure of the ability to conduct heat. Heat transfer occurs at a lower rate in materials with low thermal conductivity, and at a higher rate, in materials with high thermal conductivity. Thermal conductivity is an inherent material property that does not depend on thickness, but solely on the chemical composition of the material (1).

Although thermal conductivity depends exclusively on the material’s chemical composition, thickness can increase the resistance to heat flow (1). Thicker base layers compared to liners tend to lower the heat flow to the pulp chamber. Temperature flow should also be considered as the rate at which heat transfer from one point (hotter) to another (colder) also increases according to the temperature difference between them. The greater the temperature difference between two points, the greater the heat flow, also known as thermal diffusivity (1). Therefore, thermal conductivity as well as the thickness of base and liner materials and temperature, has a direct effect on the thermal insulation properties of liner and base materials.

During the light curing procedure, the light excites the electrons in the atoms till they make a quantum transition from electronically excited to vibrationally excited, meaning that the energy causes the whole atom to move. Then, when atoms start to collide into each other, this vibrational energy is dissipated as heat. Heat essentially means the transfer of energy between two systems (1,8-11). Although different manufacturers claim thermal insulation properties of their products, little is known regarding their effectiveness. Thus, the aim of this study was to evaluate the intra-pulpal temperature during different direct pulp capping methods using light-cured procedures. The tested hypotheses were: 1) liners are capable to provide sufficient thermal insulation for succeeding light-activated procedures; and 2) bases are capable to provide sufficient thermal insulation for succeeding light-activated procedures.

## Material and Methods

-Teeth preparation

Class I preparations 5 mm long, 4 mm wide and 4 mm deep were performed in third molar teeth leaving 0.5 mm of dentin at the pulpal floor with a 1 mm diameter of pulp exposure. First, the occlusal surface of each tooth was made flat using a grinder/polish machine (AUTOMET 250, Buehler, Lake Bluff IL, USA). Then, the roots from the teeth were sectioned to expose the pulp floor in order to acquire an intaglio view of the pulp, so that the final pulp thickness could be measured with a dental caliper.

The Class I preparations were prepared with a cylindric diamond dental bur in a rotatory hand drill (Kavo Dental, Charlotte, NC, USA) coupled to a cavity preparation machine (Odeme Dental Research, Pompano Beach, FL, USA) with three axes and dimensional control accuracy of 0.01 mm. Then, the pulp exposure was performed with a spherical diamond bur 0.01 mm in diameter. The pulp exposure location was standardized with 1 mm distance from the vestibular and mesial surfaces. For standardization purposes, the light-transmittance through the Class I preparations was evaluated to randomize the samples.

-LED curing light characterization

The mean radiant emittance (mW/cm2) for the LED curing unit used in the study (VALO Grand, Ultradent, South Jordan, UT, USA) was measured using a spectrometer-based instrument. The output power (mW) was measured with a calibrated power meter (Ophir Optronics, Har-Hotzvim, Jerusalem, Israel). The area of light emission (cm2) was measured using a digital caliper by means of five readings of the inner diameter of light tip and calculating the area using the formula (d/2)2. The radiant emittance (mW/cm2) was calculated by dividing the output power (mW) by the area of the light tip (cm2). The mean irradiance of the LED curing light used in the study was 1009 mW/cm2 ± 11.86 mW/cm2.

-Light-transmittance standardization analysis

The light-transmittance through the Class I preparations were recorded with a spectrophotometer (MARC Resin Calibrator, BlueLight Analytics, Nova Scotia, Canada). Each tooth was positioned above the input sensor of the spectrophotometer and, the light-transmittance (mW/cm2) recorded during the pre-defined exposure with the LED curing light. The light-transmittance results were used to randomize the samples. The randomization was statically evaluated using one-way ANOVA. The mean light-transmittance through the Class I preparation samples was 453 mW/cm2 ± 72.20 mW/cm2. The randomization distribution passed the Shapiro-Wilk normality test (W > 0.8; *P* > 0.6) and three different variance analysis tests, the ANOVA, the Brown-Forsythe test and the Bartlet’s test (*P* > 0.9; R2 < 0.05).

-Intrapulpal Temperature Analyses

**Figure 1:**
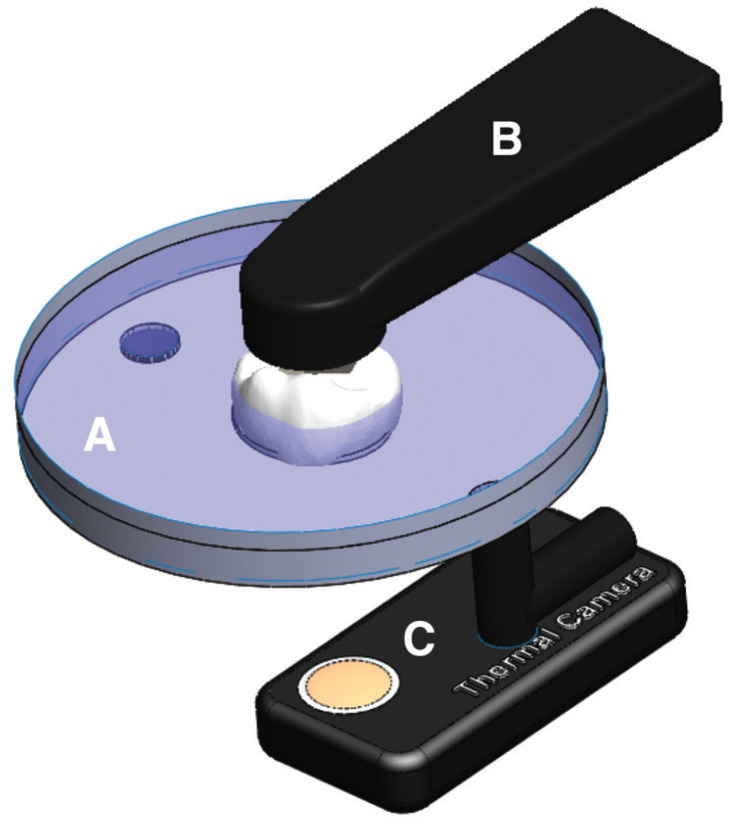
Customized oral cavity chamber simulator: (A) Circulating water bath bath with digitally controlled temperature; (B) curing light used for light-cured procedures; (C) infrared thermal camera positioned underneath the pulp chamber to live record temperature variations.

The prepared teeth were placed in a customized oral cavity chamber simulator in which the initial temperature was standardized at 36oC. The customized oral cavity chamber simulator consisted of a customized circulating water bath with the temperature being digitally controlled, in which the anatomical crown of the tooth was glued to the customized oral cavity chamber simulator with the pulp chamber baring to the outside. Figure 1 illustrates the customized oral cavity chamber simulator. An infrared camera (FLIR ONE PRO, FLIR Systems) with thermal sensitivity of <0.10oC and 150 mK was positioned underneath the pulp chamber to live record the overall temperature variations (oC) in the pulp chamber during the light-activation processes. Temperature decrease was also recorded till the temperature cooled down to the initial temperature baseline. A software (FLIR Tools) transferred the video database to a data sheet.

-Direct pulp capping procedures

The materials evaluated were: Dycal (Dentisply, York, PA, USA) and TheraCal LC (Bisco, Schaumburg, IL, USA) as liners and Biodentin (Septodent, Saint-Maur-des-Fosses, Cedex, France), Vitrebond (3M/ESPE, St. Paul, MN, USA) and Fuji IX GP (GC, Bunkyo-ku, Tokyo, Japan) as bases, followed by restoration with a bulk-fill composite (Ivoclar Vivadent, Schaan, Liechtenstein).

Dycal:

Both base and catalyst pastes were dispensed in equal volumes onto a mixing pad, and then, mixed until a totally homogeneous color was obtained. Then, a thin single layer (>1 mm in thickness) was applied exactly above the pulp exposure using a Dycal liner applicator covering all the exposed area and at least 1 mm onto dentin surrounding the exposure. After the setting time of 3 minutes, the restorative procedure was performed.

TheraCal LC:

The TheraCal LC was applied directly to the pulp exposure in a thin single layer (>1 mm in thickness) covering all the exposed area and at least 1 mm onto dentin surrounding the exposure. Then, light-cured for 20 seconds. The restorative procedure was immediately performed.

Biodentin:

The capsule was opened, and five drops from the single-dose container was dispensed into the capsule. Then, the capsule was closed, and placed on a mixing device (Ultramat, SDI, Bayswater, Australia) at a speed of 4000 rotations/min for 30 seconds. The biodentin was applied directly to the pulp floor in a thin single layer (2 mm in thickness). After the setting time of 12 minutes, the restorative procedure was performed.

Vitrebond Plus:

First the Dycal liner was applied as described before. Then, the Vitrebond Plus was dispensed onto a mixing pad, and mixed for 15 seconds. The Vitrebond Plus was applied directly to the pulp floor in a thin single layer (2 mm in thickness). Then, light-cured for 20 seconds. Hereafter, the restorative procedure was immediately performed.

Fuji IX GP:

First, the Dycal liner was applied as described before. Then, the capsule was activated by pushing the plunger until it is fully pressed. Then, the capsule was immediately placed on a mixing device (Ultramat, SDI, Bayswater, Australia) at a speed of 4000 rotations/min for 10 seconds. The Fuji IX GP was applied directly to the pulp floor in a thin single layer (2 mm in thickness). After the setting time of 6 minutes, the restorative procedure was performed.

Restorative procedures

One coat of the adhesive (Adhese Universal, Ivoclar Vivadent, Schaan, Liechtenstein) was applied for 20 seconds, gently air bloated, and light-cured for 10 seconds. Then, 2 mm or 4 mm thick layers of the bulk-fill composite were applied accordingly to each group: liners (Dycal and TheraCal), 4 mm thick layer; bases (Biodentin, Vitrebond and Fuji IX GP), 2 mm thick layer. Hereafter, the bulk-fill layer was light-cured for 10 seconds.

-Statistical Analysis 

A power analysis was conducted to determine sample size and power of at least 0.8 at a significance level of α=0.05. Randomization was statistically analyzed with Shapiro-Wilk normality test and ANOVA, Brown-Forsythe and Bartlet’s variance analysis tests. Data normality and homoscedasticity were done with Shapiro-Wilk and Lavine’s test, respectively. Statistical analyses were performed with ANOVA and Tukey’s test for multiple comparison.

## Results

**Figure 2:**
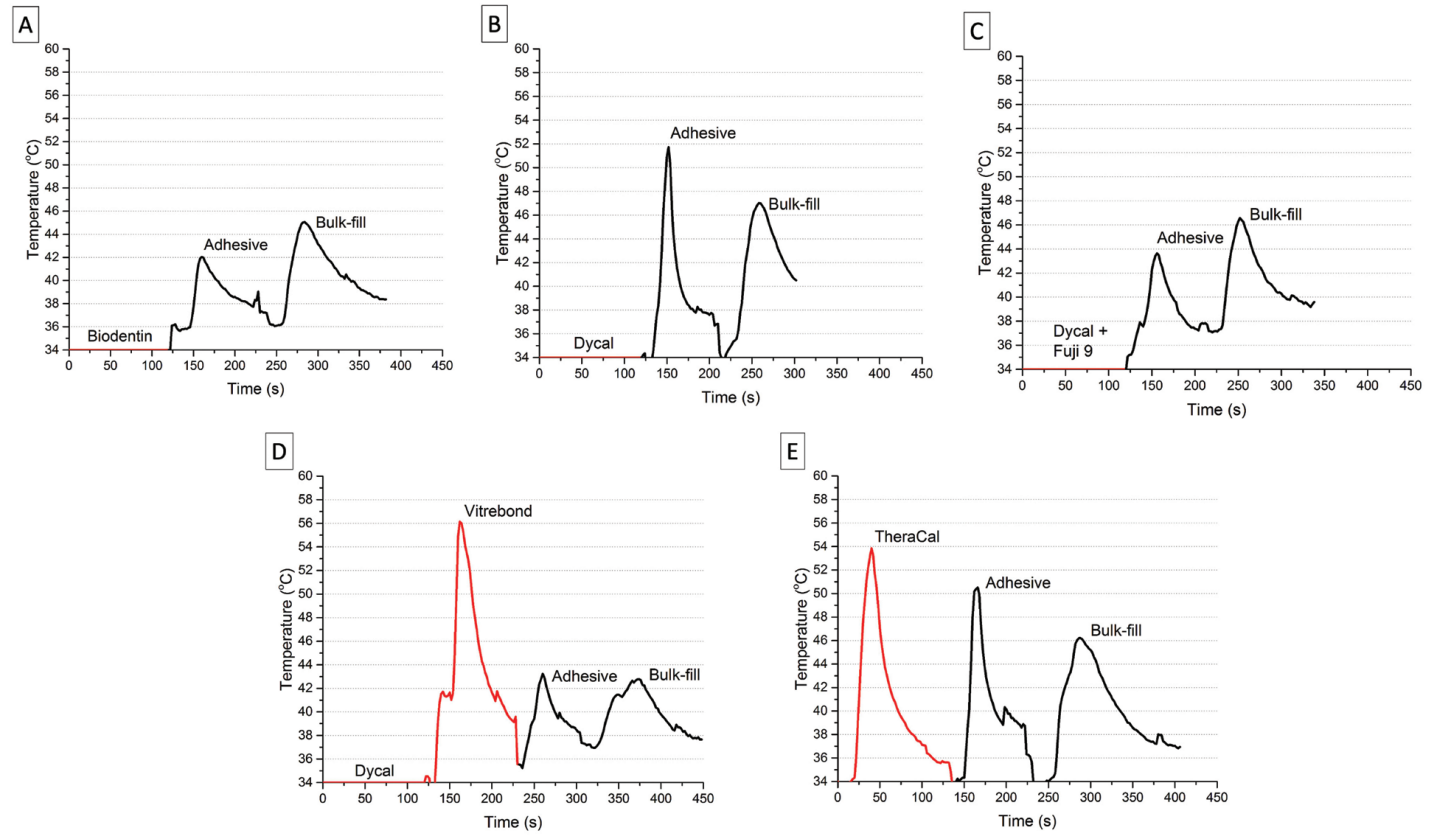
Intrapulpal temperature changes using: (A) Biodentin (Septodent); (B) Dycal (Dentsply); (C) Dycal (Denstsply) and Fuji IX (GC); (D) Dycal (Denstsply) and Vitrebond (3M); and (E) TheraCal (Bisco).

Figure 2 illustrates the intra-pulpal temperature changes during each direct pulp capping procedure. As it can be observed, the intra-pulpal temperature increased approximately 10oC to 20oC compared to the baseline temperatures during the light-curing of all liners and bases evaluated. When added as second layers, none of the tested materials could provide thermal insulation for the other following light-activated steps, such as the bonding and restorative procedures (*p*=0.25). The higher the number of procedures requiring light-activation, the longer the pulp temperature remained in those increased temperature thresholds. Biodentin was the only material tested that could provide thermal insulation following the light-activated procedures. However, it is important to point out that using bases instead of liners only, decreased the amount of heating in succeeding light-activated procedures.

## Discussion

The first hypothesis that liners would be capable of providing sufficient thermal insulation for succeeding light-activated procedures was rejected. As results indicated, both liners tested (Dycal and TheraCal LC) were not capable of providing sufficient thermal insulation for succeeding light-activated procedures. During the restorative procedures, temperature was similarly increased using both liners solely (Fig. 2). The main composition of the Dycal is the calcium hydroxide, while the TheraCal LC, a resin-modified calcium silicate designed to be light-cured. Liner’s primary purpose is to protect the pulp and promote the formation of reparative dentin bridge (2,6). However, some manufactures and research studies indicate these materials as thermal insulators (7,8). The only reason for such affirmation is because usually the thermal conductivity of most liners is lower than those of human dentin, suggesting a possible thermal insulation effect (8). Plus, the addition of a liner slightly decreases the temperature increase from succeeding light-cured procedures in comparison to when no liner is applied previously (7). However, neither were not able to prevent intra-pulpal temperature increase during the light-curing procedures. Thus, although the liners might in fact have a low thermal conductivity, it does not necessarily mean that will provide proper thermal insulation.

As can be observed in the results, during the succeeding restorative procedures, temperature was always increased from the initial temperature baseline using both liners. In addition, it is important to point out that the use a light-cured liner extended the overall time the pulp chamber temperature remained increased during the entire restorative procedure, aggravating this clinical scenario (Fig. 2).

The second hypothesis that bases would be capable to provide sufficient thermal insulation for succeeding light-activated procedures was rejected. None of the bases, such as glass ionomer (Fuji IX GP), resin-modified glass ionomer (Vitrebond Plus) or tricalcium silicate (Biodentin), were capable to provide sufficient thermal insulation for succeeding light-activated procedures. During the succeeding restorative procedures, temperature was similarly increased for all different base materials (Fig. 1). The base primary purpose is to replace missing dentin and serve as a barrier against thermal changes and chemicals and bacteria leakage (2-5). It was possible to see statistical differences in temperature rise values between when a base was placed or only liners were placed. However, the increase in temperature was still high even when bases were used before the restorative procedure.

The main composition of the Fuji IX GP is fluoroaluminosilicate glass and a polyacrylic acid (12), while the Vitrebond Plus, fluoroaluminosilicate glass in combination with dimethacrylate monomers and a polyalkenoic acid plus photoinitiators (13). The biodentin, on the other hand, contain tricalcium silicate and dicalcium silicate plus calcium carbon (9,14). Due to the differences in the composition of the different base materials tested, it is expected to have different thermal conductivities (1). Thus, this explains the small differences in temperature rise in the pulp chamber for the different tested base materials, even in similar testing conditions. Still, all liners and bases generally have lower thermal conductivity (8-10) in comparison to the tooth (15). Therefore, thickness and temperature are usually the major factors playing a hole on the thermal insulation property of these materials.

Essentially, it seems there is no ideal liner or base to avoid intra-pulpar temperature rise during light-curing procedures. Future studies should further evaluate clinical implications of using light-cured materials in deep cavities. So far, the best way to avoid temperature increase during light-curing procedures is the cooling down method using the air syringe to prevent possible overheating of the pulp tissue (16,17). In this method, directly blowing air from the air syringe to the tooth that is being light-cured helps cooling down the tooth and avoiding temperature rise.

It is well-known that any potential hazards to the pulp will depend upon the duration and exact nature of the thermal challenge. According to the most-cited study in the field, as temperature increased (above normal oral temperature) from 5.5 to 11°C, at 1 mm into dentin, the possibility of pulp necrosis increased from 15% to 60% (18). A follow-up study indicated that an 11.2°C increase would still be considered safe and not damage pulpal tissues (19). These controversial results are attribuTable to the fact that it is impossible to track the temperature changes in specific localized pulp areas, or to precisely determine how much irradiation to these areas is caused by the exposure. Overall, the heat appears not to be the major injury factor, at least in the short term. However, it is certain to play a role in postoperative inflammation or necrosis in the long term, especially when combined with other factors such as caries and high-speed bur injuries.

As could be observed in this study, even using a non-light cured liner and base before a light-cured restorative procedure was not sufficient to prevent the intra-pulpal temperature to rise and avoid possible thermal damages. Thus, for deep cavities such as simulated in this study, reducing the of procedures requiring light-activation should be indicated. Plus, alternative methods to reduce temperature rise during light-curing such as the use of air-spray should be encouraged (20).

## Conclusions

Within the limitations of this *in vitro* study, it was possible to conclude that for direct pulp capping procedures, a reduced number of procedures requiring light-activation should be indicated in order to reduce the time that the intra-pulpal temperature rises above a 10oC threshold.
